# Additional data support the role of *LINC00673* rs11655237 C>T in the development of neuroblastoma

**DOI:** 10.18632/aging.101920

**Published:** 2019-04-20

**Authors:** Yong Li, Zhen-Jian Zhuo, Haiyan Zhou, Jiabin Liu, Zan Liu, Jiao Zhang, Jiwen Cheng, Suhong Li, Haixia Zhou, Rong Zhou, Jing He, Yaowang Zhao

**Affiliations:** 1 Department of Pediatric Surgery, Hunan Children’s Hospital, Changsha 410004 Hunan, China; 2 Department of Pediatric Surgery, Guangzhou Institute of Pediatrics, Guangzhou Women and Children’s Medical Center, Guangzhou Medical University, Guangzhou 510623 Guangdong, China; 3 Department of Pathology, Xiang-ya School of Medicine, Central South University, Changsha 410013 Hunan, China; 4 Department of Pediatric Surgery, The First Affiliated Hospital of Zhengzhou University, Zhengzhou 450052 Henan, China; 5 Department of Pediatric Surgery, The Second Affiliated Hospital of Xi’an Jiaotong University, Xi’an 710004 Shaanxi, China; 6 Department of Pathology, Children Hospital and Women Health Center of Shanxi, Taiyuan 030013 Shannxi, China; 7 Department of Hematology, The Second Affiliated Hospital and Yuying Children’s Hospital of Wenzhou Medical University, Wenzhou 325027 Zhejiang, China; 8 Science and Education Section, Hunan Children’s Hospital, Changsha 410004 Hunan, China; * Equal contribution

**Keywords:** neuroblastoma, *LINC00673*, polymorphism, susceptibility

## Abstract

Neuroblastoma is the most frequently diagnosed neural tumor of childhood. Abnormal function of the long intergenic non-coding RNA (lincRNA) *LINC00673* has been implicated in various human malignancies. Genome-wide association studies revealed the *LINC00673* rs11655237 C>T polymorphism to be associated with the risk of neuroblastoma, though the effect was not well defined, in part due to the small sample size in our earlier study. Herein, we verified the impact of *LINC00673* rs11655237 C>T on the risk of neuroblastoma in 700 cases and 1516 controls from six centers in China. After pooling all enrolled patients, we observed a significant association between *LINC00673* rs11655237 C>T and risk of neuroblastoma (TT vs. CC: adjusted odds ratio [OR]=1.58, 95% confidence interval [CI]=1.06–2.35, *P*=0.024; additive model: adjusted OR=1.20, 95% CI=1.03–1.39, *P*=0.020; recessive model: adjusted OR=1.50, 95% CI=1.02–2.22, *P*=0.040). Stratification analysis revealed a significant relationship between rs11655237 CT/TT and neuroblastoma risk in subgroups of males, patients whose tumor originated in the adrenal gland, and patients with clinical stage IV disease. These findings add new evidence of the importance of *LINC00673* rs11655237 C>T to the risk of developing neuroblastoma.

## INTRODUCTION

Neuroblastoma is the most commonly seen solid childhood tumor outside the brain [1, 2]. It generally results from a differentiation failure of neural crest cell precursors [3]. Despite being relatively rare (nearly 8% of all pediatric cancer diagnoses), neuroblastoma disproportionately contributes to about 15% of all childhood cancer-related mortality [4–6]. Outcomes greatly vary among neuroblastoma patients, spanning a range from spontaneous regression with little or no treatment to progression despite aggressive, multimodal therapy [7]. At present, the five-year survival rate for patients with metastatic high-risk neuroblastoma is less than 50% after multimodal therapy [8].

Neuroblastoma is a heterogenous cancer influenced by both environmental and genetic factors. Previous studies have shown that children and child-bearing women exposed to certain kinds of environmental factors are more likely to develop neuroblastoma [9–11]. In addition, genetic factors, such as *ALK* [12, 13] and *PHOX2B* [14, 15] mutations, have also been implicated in neuroblastoma progression. Recent progress in genome-wide association studies (GWASs) has helped to identify single nucleotide polymorphisms (SNPs) that contribute to neuroblastoma risk. So far, it appears that SNPs in *LMO1* [16], *TP53* [17], *LIN28B* [18], *BARD1* [19], *DUSP12* [20], *HACE1* [18], *NEFL* [21] and *CDKN1B* [22] contribute to the risk of neuroblastoma. However, the currently identified variants do not fully account for the etiology of neuroblastoma.

Long intergenic non-coding RNAs (lincRNAs) are a class of transcripts that are longer than 200 nucleotides but do not code for a protein [23]. LincRNAs modulate cellular activities through multiple mechanisms, including transcriptional regulation, epigenetic regulation, imprinting, genome rearrangement, and chromatin modification [24]. Over the past decade, a variety of lincRNAs have been shown to play key roles in human disorders, including cancers [25, 26]. For example, *LINC00673* (OMIM No. 617079) has been implicated in the development and prognosis of several malignancies [27–29], and polymorphism in *LINC00673*, rs11655237 C>T (also reported as G>A elsewhere), was identified as being significantly associated with susceptibility to pancreatic cancer [30]. In an earlier investigation, we observed a significantly increased risk of neuroblastoma in subjects carrying the T allele of *LINC00673* rs11655237 [31]. This prompted us to further evaluate the relationship between *LINC00673* rs11655237 C>T and the risk of neuroblastoma in a larger sample.

## RESULTS

### Association between the *LINC00673* rs11655237 C>T and neuroblastoma risk

The baseline characteristics of the neuroblastoma patients and controls are summarized in Supplementary Table 1 and in our previously published articles [32–35]. There were no significant differences in the age or gender distributions between the cases and controls, which indicates that the frequency matching was adequate. The genotype frequencies of *LINC00673* rs11655237 C>T for all the subjects and their contributions to neuroblastoma risk are listed in Table 1. The observed genotype distributions of the rs11655237 C>T polymorphism among the controls were consistent with the Hardy-Weinberg equilibrium (HWE) for both the Hunan subjects (HWE=0.268) and the combined patient sample (HWE=0.824). We detected no significant association between *LINC00673* rs11655237 C>T and neuroblastoma risk among the Hunan subjects. After pooling all the subjects, however, we found that those harboring the T allele were significantly more likely to develop neuroblastoma (TT vs. CC: adjusted odds ratio [OR]=1.58, 95% confidence interval [CI]=1.06–2.35, *P*=0.024; additive model: adjusted OR=1.20, 95% CI=1.03–1.39, *P*=0.020; recessive model: adjusted OR=1.50, 95% CI=1.02–2.22, *P*=0.040).

**Table 1:** *LINC00673* rs11655237 C>T polymorphism and neuroblastoma susceptibility.

**Genotype**	**Cases (N=698)**	**Controls (N=1516)**	***P*^a^**	**Crude OR (95% CI)**	***P***	**Adjusted OR (95% CI)^b^**	***P*^b^**
Hunan subjects (HWE=0.268)							
CC	101 (62.35)	165 (61.11)		1.00		1.00	
CT	54 (33.33)	96 (35.56)		0.92 (0.61–1.39)	0.690	0.91 (0.60–1.38)	0.648
TT	7 (4.32)	9 (3.33)		1.27 (0.46–3.52)	0.645	1.57 (0.56–4.39)	0.395
Additive			0.804	0.99 (0.70–1.40)	0.965	1.02 (0.72–1.45)	0.906
Dominant	61 (37.65)	105 (38.89)	0.798	0.95 (0.64–1.42)	0.799	0.96 (0.64–1.43)	0.830
Recessive	155 (95.68)	261 (96.67)	0.599	1.31 (0.48–3.59)	0.600	1.62 (0.58–4.50)	0.355
Combined (HWE=0.824)							
CC	401 (57.45)	935 (61.68)		1.00		1.00	
CT	252 (36.10)	513 (33.84)		1.15 (0.95–1.39)	0.163	1.14 (0.94–1.38)	0.175
TT	45 (6.45)	68 (4.49)		**1.54 (1.04**–**2.29)**	**0.031**	**1.58 (1.06**–**2.35)**	**0.024**
Additive			0.057	**1.19 (1.03**–**1.38)**	**0.022**	**1.20 (1.03**–**1.39)**	**0.020**
Dominant	297 (42.55)	581 (38.32)	0.059	1.19 (0.99–1.43)	0.059	1.19 (0.99–1.43)	0.059
Recessive	653 (93.55)	1448 (95.51)	0.051	1.47 (0.996–2.16)	0.053	**1.50 (1.02**–**2.22)**	**0.040**

OR, odds ratio; CI, confidence interval; HWE, Hardy-Weinberg equilibrium.

^a^χ^2^ test for genotype distributions between neuroblastoma cases and cancer-free controls.

^b^Adjusted for age and gender.

### Stratification analysis

To identify vulnerable subgroups, we stratified the subjects based on selected variables (Table 2). This enabled us to detect a significant association between rs11655237 CT/TT and neuroblastoma risk in males (adjusted OR=1.34, 95% CI=1.05–1.71, *P*=0.019). Taking sites of tumor origin into consideration, we observed that carriers of the rs11655237 CT/TT genotype were more likely to develop tumors originating in the adrenal gland (adjusted OR=1.36, 95% CI=1.02–1.81, *P*=0.039). Moreover, carriers of the CT/TT genotypes had a significantly higher risk of INSS clinical stage IV disease than carriers of the CC genotype (adjusted OR=1.50, 95% CI=1.11–2.04, *P*=0.009).

**Table 2:** Stratified results for *LINC00673* rs11655237 C>T polymorphism and neuroblastoma susceptibility in the combined patient sample.

**Variables**	**rs11655237 (cases/controls)**		**OR (95% CI)**	***P***	**AOR (95% CI)^a^**	***P*^a^**
	**CC**	**CT/TT**				
Age, month						
≤18	161/378	112/237	1.11 (0.83–1.48)	0.484	1.12 (0.83–1.49)	0.459
>18	240/557	185/344	1.25 (0.99–1.58)	0.064	1.25 (0.99–1.58)	0.065
Gender						
Females	182/393	125/263	1.03 (0.78–1.35)	0.854	1.03 (0.78–1.35)	0.853
Males	219/542	172/318	**1.34 (1.05**–**1.71)**	**0.019**	**1.34 (1.05**–**1.71)**	**0.019**
Sites of origin						
Adrenal gland	117/935	98/581	**1.35 (1.01**–**1.80)**	**0.042**	**1.36 (1.02**–**1.81)**	**0.039**
Retroperitoneal	137/935	101/581	1.19 (0.90–1.57)	0.227	1.19 (0.90–1.57)	0.218
Mediastinum	107/935	70/581	1.05 (0.77–1.45)	0.752	1.04 (0.76–1.44)	0.790
Others	38/935	22/581	0.93 (0.55–1.59)	0.796	0.93 (0.55–1.59)	0.795
Clinical stages						
I	123/935	93/581	1.22 (0.91–1.62)	0.183	1.22 (0.91–1.62)	0.187
II	78/935	51/581	1.05 (0.73–1.52)	0.786	1.04 (0.72–1.50)	0.833
III	88/935	46/581	0.84 (0.58–1.22)	0.362	0.84 (0.58–1.21)	0.345
IV	101/935	94/581	**1.50 (1.11**–**2.02)**	**0.008**	**1.50 (1.11**–**2.04)**	**0.009**
4s	6/935	10/581	2.68 (0.97–7.42)	0.057	2.80 (0.98–7.95)	0.054
I+II+4s	201/935	144/581	1.15 (0.91–1.46)	0.241	1.15 (0.91–1.46)	0.248
III+IV	189/935	140/581	1.19 (0.94–1.52)	0.155	1.19 (0.93–1.52)	0.165

OR, odds ratio; CI, confidence interval; AOR, adjusted odds ratio.

^a^Adjusted for age and gender, omitting the corresponding stratification factor.

### False-positive report probability analysis

In a false-positive report probability analysis (Table 3) at a prior probability level of 0.1, a significant association with neuroblastoma risk was noteworthy for males and clinical stage IV patients carrying CT/TT genotypes. At a prior probability level of 0.25, the increased neuroblastoma risk remains noteworthy in carriers of the rs11655237 TT and rs12587 CT/TT genotypes for the male, adrenal tumor, and stage IV subgroups.

**Table 3:** False-positive report probability analysis for significant findings in the combined patient sample.

**Genotype**	**Crude OR (95% CI)**	***P*^a^**	**Statistical power^b^**	**Prior probability**				
				**0.25**	**0.1**	**0.01**	**0.001**	**0.0001**
TT vs. CC	1.54 (1.04–2.29)	0.031	0.469	**0.166**	0.374	0.868	0.985	0.998
CT/TT vs. CC								
Males	1.34 (1.05–1.71)	0.019	0.823	**0.063**	**0.168**	0.690	0.957	0.996
Adrenal gland	1.35 (1.01–1.80)	0.042	0.769	**0.141**	0.329	0.844	0.982	0.998
Stage IV	1.50 (1.11–2.02)	0.008	0.509	**0.046**	**0.127**	0.615	0.941	0.994

OR, odds ratio; CI, confidence interval.

^a^Chi-square test was used to calculate the genotype frequency distributions.

^b^Statistical power was calculated using the number of observations in the subgroup and the OR and *P* values in this table.

## DISCUSSION

In the present study, we further evaluated the association between *LINC00673* rs11655237 C>T and the risk of neuroblastoma using a larger sample. Our results indicate that *LINC00673* rs11655237 C>T is significantly associated with increased neuroblastoma susceptibility, which further highlights the important contribution of *LINC00673* rs11655237 C>T to the risk of neuroblastoma in Chinese children.

*LINC00673* is located on chromosome 17q24.3, which exhibits a high frequency of loss of heterozygosity [36]. In 2011, a rs11655237 C>T variant was first documented by Cabiliet et al. [37]. Later, a GWAS showed rs11655237 C>T to be associated with pancreatic cancer susceptibility [30]. A subsequent study by Zheng et al. [38] confirmed the relationship between rs11655237 and the risk of pancreatic ductal adenocarcinoma (PDAC) in a Chinese population and shed light on the molecular mechanism by which the polymorphism confers PDAC risk. They found that the C-to-T shift at rs11655237 creates a target site for miR-1231 binding and interferes with ubiquitination and degradation of PTPN11. They also demonstrated that the rs11655237 T allele can impair *LINC00673* activity, thereby triggering SRC-ERK oncogenic signaling and ultimately resulting in a higher risk for developing PDAC. Intriguingly, *LINC00673* functions as a tumor suppressor or promoter in different cancer types. Huang et al. [29] found that *LINC00673* is upregulated in gastric cancer and is associated with a poor prognosis. Investigation into the mechanism suggested that *LINC00673* is activated by SP1 and exerts oncogenic effects in part through interaction with LSD1 and EZH2. Lu et al. [39] also identified *LINC00673* as an oncogenic mediator in non-small cell lung cancer. They demonstrated that *LINC00673* promotes non-small cell lung cancer cell proliferation, migration, invasion, and epithelial mesenchymal transition by sponging miR-150-5p. In addition, research conducted by Yu et al. [40] showed that *LINC00673* is highly expressed in human tongue squamous cell carcinoma and correlates with a poor prognosis. Up to now, however, the possible activity of *LINC00673* in neuroblastoma remained unexplored.

Considering the important functional role of *LINC00673* in malignancies, we conducted the first case-control study to investigate the association between *LINC00673* rs11655237 C>T polymorphism and neuroblastoma risk in a Chinese population [31]. In that two-center study with 393 neuroblastoma patients and 812 healthy controls, we found that *LINC00673* rs11655237 C>T polymorphism confers a high risk of neuroblastoma. More recently, the epidemiological role of *LINC00673* rs11655237 C>T was examined within the context of cervical cancer [41]. Wang et al. observed that rs11655237 contributes significantly to a higher susceptibility to cervical cancer in a Chinese population, which they speculated may reflect decreased *LINC00673* expression caused by the T allele. In the present study, we further investigated the association between *LINC00673* rs11655237 C>T and neuroblastoma risk using a larger sample from multiple centers in China. Unexpectedly, we did not detect a significant contribution of *LINC00673* rs11655237 C>T to neuroblastoma risk in the patient sample from Hunan. However, significant associations were revealed after combining the patients from all six centers. This finding is biologically plausible, as the relatively small sample size and low-penetrance of a single polymorphism could account for the null association. Interestingly, the impact of *LINC00673* rs11655237 C>T on susceptibility to neuroblastoma was similar to that for pancreatic cancer. This suggests *LINC00673* rs11655237 C>T may confer neuroblastoma and pancreatic cancer risk via similar molecular mechanisms.

This is a pioneer multi-center case-control study examining the contribution of *LINC00673* rs11655237 C>T to neuroblastoma risk. The study has several minor limitations. First, despite our relatively large sample size, the numbers in some stratified analyses were not sufficient to provide convincing statistical power. Second, because all the included subjects were from hospitals located in China, inherent selection bias cannot be completely excluded and the conclusions drawn may not be representative for other populations. Third, the single SNP analyzed here is far from enough to elucidate the full spectrum of neuroblastoma etiologies. Analysis of additional SNPs and use of additional methods of analysis (e.g., find mapping) will help to identify more neuroblastoma-associated loci [42]. Finally, SNP-SNP and SNP-environmental factor interaction analyses are absent. The role of *LINC00673* rs11655237 C>T in neuroblastoma may be modified by such interactions.

In summary, our findings support the notion that *LINC00673* rs11655237 C>T polymorphism may predispose one to neuroblastoma. That conclusion needs to be by further substantiated by research exploring the possible mechanisms by which *LINC00673* rs11655237 C>T could modulate neuroblastoma risk.

## MATERIALS AND METHODS

### Study subjects

In brief, we carried out a six-hospital-based case-control study in China. A total of 162 cases and 270 controls from Hunan Children’s Hospital were freshly genotyped in the present study. The other study subjects have been described in detail elsewhere [32, 34, 43]. In all, 700 cases and 1516 controls were recruited from six regional hospitals in China (275 cases and 531 controls from Guangzhou Women and Children’s Medical Center, 76 cases and 186 controls from The Second Affiliated Hospital of Xi’an Jiaotong University, 36 cases and 72 controls from The Second Affiliated Hospital and Yuying Children’s Hospital of Wenzhou Medical University, 118 cases and 281 controls from The First Affiliated Hospital of Zhengzhou University, 33 cases and 176 controls from Children’s Hospital of Shanxi, and 162 cases and 270 controls from Hunan Children’s Hospital). Eligible controls, frequency-matched to cases with respect to age, sex, and study center, were recruited from the same region as the cases during the same period. Informed consent was obtained from all subjects or their guardians, and the study was approved by the institutional review boards of all the participating hospitals.

### Genotyping

DNA was extracted from blood samples collected from the study participants. For genotyping, TaqMan methodology was according to the manufacturer’s instructions [44–47]. For quality control, technicians were blind to the status of the samples, and 10% of the samples were genotyped twice to ensure genotyping accuracy. The results for the random duplicate samples were 100% concordant.

### Statistical analysis

The χ2 test was used to determine whether the observed genotype frequencies among the control subjects were in line with the Hardy-Weinberg equilibrium. The χ2 test was also adopted to compare differences in selected demographic variables and genotype frequencies between the cases and controls. Multivariable logistic regression analysis adjusted for age and gender was conducted to determine the association between rs11655237 C>T and neuroblastoma risk based on ORs and their 95% CIs. Values of *P*<0.05 were considered significant. We also performed a false-positive report probability analysis, as described previously [48–50]. SAS ver. 9.1 (SAS Institute Inc., Cary, NC, USA) was used for all statistical analyses.

## Supplementary Material

Supplementary Table 1
