# Supplementary material for: Additional data support the role of LINC00673 rs11655237 C>T in the development of neuroblastoma
Source: Aging (Albany NY). 2019 Apr 20;11(8):2369–77. doi: 10.18632/aging.101920 (PMC6520001; doi:10.18632/aging.101920)
Supplement: Supplementary Table 1 [file aging-11-101920-s001.docx]

SUPPLEMENTARY Material

| Supplementary Table 1. Frequency distribution of selected characteristics in neuroblastoma cases and cancer-free controls. | | | | | | | | | | |
| --- | --- | --- | --- | --- | --- | --- | --- | --- | --- | --- |
| **Variables** | **Combined subjects** | | | | | **Hunan province** | | | | |
|  | **Cases (n=700)** | | **Controls (n=1516)** | | ***P*^a^** | **Cases (n=162)** | | **Controls (n=270)** | | ***P*^a^** |
|  | **No.** | **%** | **No.** | **%** |  | **No.** | **%** | **No.** | **%** |  |
| Age range, month | 0.00–132.00 | | 0.004–156.00 | | 0.525 | 0.033–130.00 | | 0.033–101.00 | | 0.322 |
| Mean ± SD | 33.17±28.14 | | 30.67±25.20 | |  | 34.56±30.30 | | 27.81±19.83 | |  |
| ≤18 | 274 | 39.14 | 615 | 40.57 |  | 69 | 42.59 | 102 | 37.78 |  |
| >18 | 426 | 60.86 | 901 | 59.43 |  | 93 | 57.41 | 168 | 62.22 |  |
| Gender |  |  |  |  | 0.796 |  |  |  |  | 0.842 |
| Female | 307 | 43.86 | 656 | 43.27 |  | 79 | 48.77 | 129 | 47.78 |  |
| Male | 393 | 56.14 | 860 | 56.73 |  | 83 | 51.23 | 141 | 52.22 |  |
| INSS stages |  |  |  |  |  |  |  |  |  |  |
| I | 216 | 30.86 | / | / |  | 48 | 29.63 | / | / |  |
| II | 129 | 18.43 | / | / |  | 22 | 13.58 | / | / |  |
| III | 134 | 19.14 | / | / |  | 54 | 33.33 | / | / |  |
| IV | 196 | 28.00 | / | / |  | 37 | 22.84 | / | / |  |
| 4s | 16 | 2.29 | / | / |  | 1 | 0.62 | / | / |  |
| NA | 9 | 1.29 | / | / |  | / | / | / | / |  |
| Sites of origin |  |  |  |  |  |  |  |  |  |  |
| Adrenal gland | 215 | 30.71 | / | / |  | 31 | 19.14 | / | / |  |
| Retroperitoneal region | 240 | 34.29 | / | / |  | 78 | 48.15 | / | / |  |
| Mediastinum | 177 | 25.29 | / | / |  | 36 | 22.22 | / | / |  |
| Other region | 60 | 8.57 | / | / |  | 17 | 10.49 | / | / |  |
| NA | 8 | 1.14 |  |  |  | / | / | / | / |  |
| SD, standard deviation; NA, not available.  ^a^Two-sided χ^2^ test comparing distributions between neuroblastoma cases and cancer-free controls. | | | | | | | | | | |
